# Analysis of school support: Systematic literature review of core Chinese- and English-language journals published in 2000–2021

**DOI:** 10.3389/fpsyg.2022.933695

**Published:** 2022-08-08

**Authors:** Jun Li, Ziao Hu, Ling Pan

**Affiliations:** ^1^School of Finance and Economics, Hainan Vocational University of Science and Technology, Haikou, China; ^2^Department of Education Management, Chinese International College, Dhurakij Pundit University, Bangkok, Thailand

**Keywords:** school support, systematic literature review, definition, measurement tools, articles

## Abstract

School support is of great significance to students' academic quality and overall physical and psychological development. However, there is still ambiguity in the English and Chinese studies on the concept and measurement tools of school support. The data for this study were sourced from the literature on school support included in the China National Knowledge Network (CNKI) and Web of Science (WOS) from 2000 to 2021. A systematic literature review was conducted through literature inclusion and data extraction according to the PRISMA guidelines. Finally, 36 core-journal articles with high academic reference value and authority are identified, including seven in Chinese and 29 in English. The following results were obtained: (1) Related research in both Chinese and English showed an overall increasing trend. (2) The concepts and measurement tools of school support were not clear, and most studies used concepts and measurement tools of “social support” or “school climate” as substitutes for school support. (3) Most of the previous studies were based on social support theory, ecological system theory, and school climate theory. (4) The research mainly adopts quantitative research methods and focuses on special student populations or students below the high school level. Overall, previous studies indicated that school support has a positive impact on student development. Therefore, future research should be broadly extended to the knowledge system in higher education. On the basis of clarifying the concept of school support, it is necessary to try to develop and validate school support measurement tools with great reliability, validity, and general applicability to provide a practical reference for educators around the world.

## Introduction

According to ecological system theory, school is one of the most proximal and essential factors influencing individual development at the microsystem level (Bronfenbrenner, 1977, 1992; Axlund McBride and Lott, 2015). As a place where adolescents spend more than half of their adequate time every day, school plays an important role in the holistic development and healthy growth of adolescents (Eccles and Roeser, 2011; Tang et al., 2013). American scholars Sugai and Horner pointed out that positive behavioral support at the school level was conducive to the overall academic and social development of students. In particular, it has an effective preventive effect on students with severe problem behaviors (Warren et al., 2006; Sugai and Horner, 2009; Yang and Li, 2016). Based on previous research, school support has been shown to be a vital factor in the healthy development of adolescents (Gregory et al., 2010), which can not only buffer the adverse effects of academic stress (Torsheim and Wold, 2001) and enhance creativity and individual academic performance (Zhang et al., 2020) but also positively affect academic engagement, school participation, and emotional engagement (Bottiani et al., 2016; Yang et al., 2020). Therefore, research on school support is of great significance and value.

There are different views on the definition and measurement of school support depending on researchers' respective research scenarios. For example, Yang et al. (2020) defined school support as a combination of internal school values, school climate, and interpersonal relationships that reflects the quality of school life, and the school climate scale that measures the degree of school support for secondary school students with special needs. This definition and measurement have also been used in recent studies on school support (Fang et al., 2016; Zhang et al., 2020). Differently, Cornell et al. (2015) claimed that school support was only one aspect of school climate. Bottiani et al. (2016) agreed with Cornell et al. (2015) that school support was an emerging theoretical construct aimed at meeting adolescents' needs for belonging, competence, and autonomy. Based on this, they explored a 3D model for measuring school support (school equity, school care, and high expectations).

In addition, previous research argued that the concept of school support was derived from the application and development of organizational support theory in education (Eisenberger et al., 1997; Hu and Liu, 2019; Deng et al., 2020). In the complex social organization of schools, teachers and peers are two different sources of support (Moreira and Lee, 2020). Therefore, the social support from school (teacher and peer support) and autonomy support explain school support from the school level (Moreira and Lee, 2020). Other studies identified teachers and peers (classmates) as the primary sources of social support in schools. School support was measured through the integration of teacher support and peer support scales. In this view, school support is defined as the level of support students feel from teachers and peers (Torsheim and Wold, 2001; Cupito et al., 2016; Zhang et al., 2020). Besides, some studies equated teacher support with school support. For example, in Corprew and Cunningham's (2011) study of African American male students aged 13–18, they pointed out that school support was the support students perceived from teachers and administrators. Among them, four teacher support items in the social support scale were used to measure school support. Moreover, based on the findings from recent interviews with black adolescents aged 12–18 on academic adjustment and mental health issues during the COVID-19 pandemic (Parker et al., 2021), school support specifically includes instrumental and emotional support from school staff and teachers.

For related domestic research, Luo and Xiang (2011) developed the “School Support System Questionnaire for Students with Cerebral Palsy” to investigate the 6–18-year-old students with cerebral palsy. The questionnaire contains five dimensions, namely environmental support, teacher support, peer support, examination and assessment support, and classroom teaching support. Ke et al. (2019) developed a school support scale (including two dimensions of course study resources and venue activity resources) to explore the impact of school support on the personalized growth of 5th-grade students in primary school.

In conclusion, there is inconsistency in the definition of school support, and the measurement tools also lack uniform and universal scales. Therefore, this study systematically combed Chinese and English literature from 2000 to 2021 in two highly acknowledged academic databases in China and around the globe, namely China National Knowledge Infrastructure (CNKI) and Web of Science (WOS), aiming to comprehensively understand the knowledge system, research status, and theoretical bases of school support and clarify its concept. Specifically, the following four research questions were focused on the following:

(1) What is the current state of research on school support?
(2) How do related studies define school support?
(3) What is the theoretical basis of school support?
(4) What are the measurement tools of school support?

## Method

The present systematic literature review (SLR) was conducted following the guidelines of the Preferred Reporting Items for Systematic Reviews and Meta-Analysis (Moher et al., 2009). SLR is a method of assessing and clarifying all available studies related to a specific research question, topic, or area of interest (Brereton et al., 2007), with the advantages of comprehensiveness, rigor, and transparency. In more detail, straightforward research questions, comprehensive search strategies, explicit literature criteria, a high-quality assessment process, comprehensive data analysis, and reliable research results can effectively overcome the subjectivity and bias of traditional research methods (Sutherland, 2004).

### Eligibility criteria

Following PRISMA guidance on Eligibility Criteria (Moher et al., 2009), this study focused on the current status, theoretical basis, definitions (concepts), and measurement tools for school support in Chinese and English core-journal articles.

#### Inclusion criteria

(1) Core-journal articles of authority and research value published in Chinese or English.
(2) Published between January 1, 2000 and December 31, 2021.
(3) Journal-type was academic articles with school support as the research theme, and the research participants were students.
(4) Other relevant articles unearthed from the reference list can address this research question.

#### Exclusion criteria

(1) Articles without explicit publication date restrictions.
(2) Articles published in languages other than Chinese and English.
(3) Documents identified as “gray literature” (Hopewell et al., 2007), such as degree dissertations, conference papers, reviews, newspapers, government policy papers, reports, videos, and unpublished data.
(4) Articles that did not focus on school support or serve only as background or advice.

### Data sources and search strategy

Based on the research questions, this study selected the CNKI (Beijing, PRC; https://www.cnki.net/), a highly recognized Chinese academic website, and WOS (Clarivate Analytics, Philadelphia, USA), a well-acknowledged international academic database, to ensure the quality and authority of the literature samples. A total of 1,035 documents in Chinese and 549 documents in English were retrieved with the time range from January 1, 2000 to December 31, 2021 and the theme of “school support.” The reason for this time restriction was the amount of research on school support in Chinese documents before 2000 was very limited. Furthermore, some references to the selected articles were reverse searched for more articles that contributed to this research topic.

### Screening strategy and data collection

**Figure 1:**
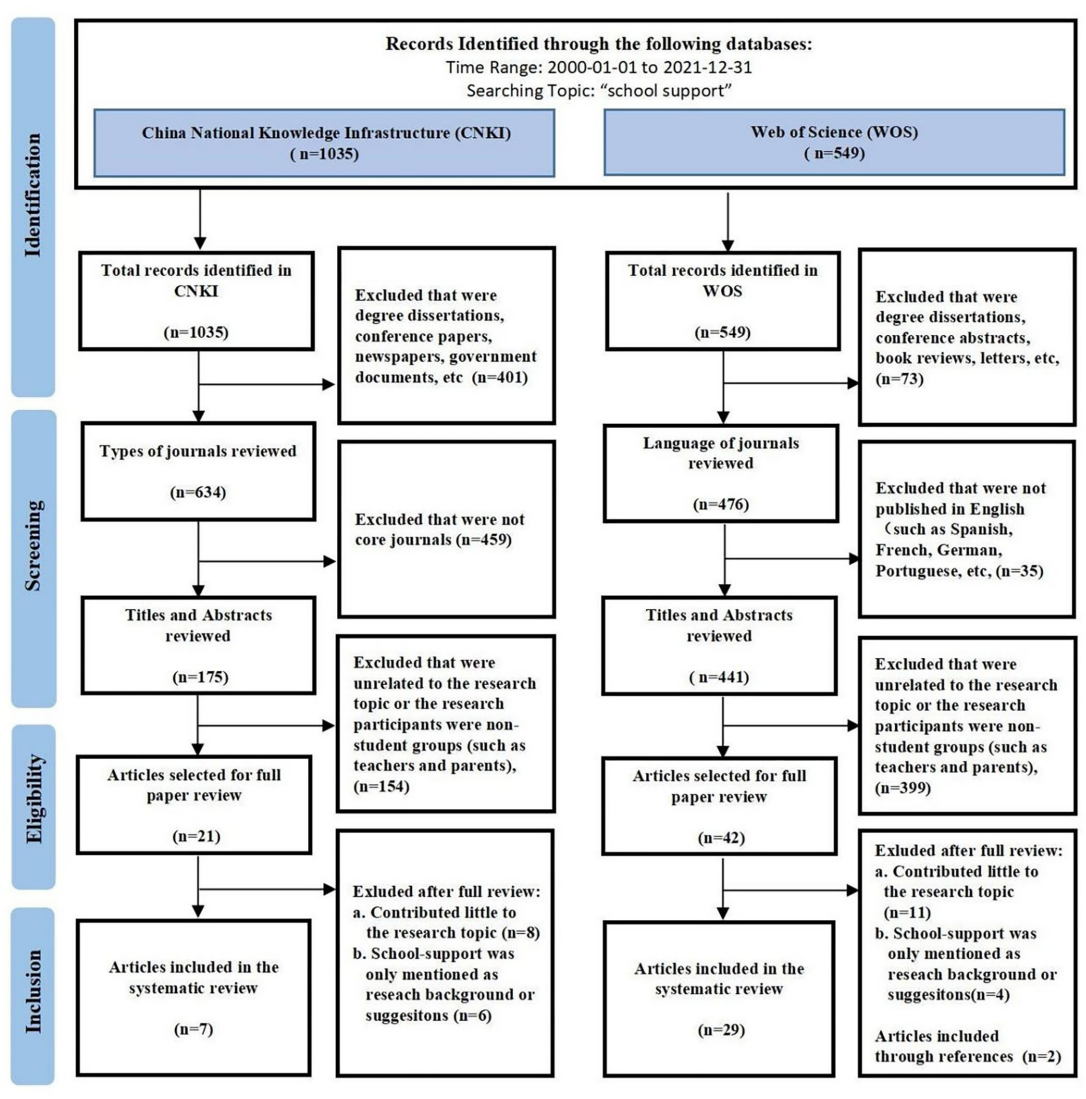
Flow chart based on PRISMA guidelines.

To ensure the validity of the sample literature, authors 1 (Li) and 2 (Hu) independently screened and collected data according to uniform selection criteria. Any disagreements between the two authors were discussed with author 3 (Pan). The PRISMA flowchart during the selection process (Moher et al., 2009) is shown in Figure 1, detailing the number of documents retained and excluded at each step as well as the corresponding reasons.

First, 1,035 documents in Chinese and 549 documents in English retrieved from the preliminary search were systematically screened.

The screening strategies for Chinese literature were as follows:

(1) In the first step, the document type was an academic article. Six hundred thirty-four articles were retained and 401 documents, such as degree dissertations, conference papers, newspapers, and government documents, were excluded.
(2) In the second step, only core journals were considered to maintain academic authority. One hundred seventy-five articles were retained, and 459 articles published in general journals were excluded. It should be noted that the journals on CNKI are divided into core journals and general journals, among which core journals are formally rated by Chinese research institutions with academic authority. Therefore, articles published in such journals have more academic reference value.

According to the screening strategy, 175 Chinese core-journal articles meeting the criteria were obtained and 860 documents were excluded.

The screening strategies for English literature were as follows:

(1) In the first step, the document type was an academic article. Four hundred seventy-six articles were retained and 73 documents, such as degree dissertations, conference abstracts, book reviews, and letters, were excluded.
(2) In the second step, 441 articles published in English were retained and 35 articles published in Spanish, French, German, Portuguese, and other languages were excluded.

According to the screening strategy, 441 English core-journal articles that met the criteria were obtained and 108 documents were excluded.

Second, the remaining 175 Chinese core-journal articles and 441 English core-journal articles were reviewed in detail according to the eligibility criteria. By reading the titles and abstracts, Author 1 (Li) and Author 2 (Hu) independently removed 154 Chinese articles and 399 English articles whose participants were non-student groups, such as teachers, parents, or who were not relevant to the topic of this study. Finally, 21 Chinese and 42 English articles were retained without disagreement between author 1 (Li) and Author 2 (Hu).

In total, 21 Chinese and 42 English articles were loaded into two separate folders through the EndNote X8 reference management software (Thomson Reuters, New York City, NY, USA). Duplicates were queried, and the full articles were exported. The full text that cannot be downloaded through EndNote X8 was downloaded manually. Through the further full-text intensive reading of 21 Chinese articles and 42 English articles, articles that did not significantly contribute to this research question were excluded. A total of 7 Chinese articles and 27 English articles were selected. Finally, the reference lists of the selected English articles were reverse searched so that two valuable articles (Jia et al., 2009; Cornell et al., 2015) that were also included in the review were obtained. In this session, authors 1 (Li) and 2 (Hu) disputed the inclusion and exclusion of four of the articles, which then was discussed with author 3 (Pan). Three authors used the Quality Assessment Tool for Observational Cohort and Cross-Sectional Studies (National Heart Lung Blood Institute, 2019) to examine the content and agreed on the final sample collection (seven Chinese articles and 29 English articles).

### Data analysis

The search, collection, and sample analysis for this study were conducted during 20–28 June 2022. The Quality Assessment Tool developed by the National Institutes of Health (National Heart Lung Blood Institute, 2019) was used to assess quality and risk of bias. In recent years, this tool has been widely used in SLR studies to assess the quality of articles and the risk of bias in studies (Carbia et al., 2018; Amit et al., 2020; Putra et al., 2020; Costa Cordella et al., 2021). Similar to article screening and data extraction, quality assessment was performed independently by two authors. Furthermore, any disputes were discussed with a third author.

Statistical analysis and content analysis were performed on the sample. Through the statistical analysis of 1,035 documents in Chinese and 549 documents in English retrieved from the initial search, the research trends supported by the school from 2000 to 2021 were visually reflected. For the authoritatively indexed 7 Chinese core-journal articles and 29 English core-journal articles, analysis of content with general characteristics and evaluation of research quality were carried out to explore definitions, theoretical bases, and measurement tools of school support. In addition, the findings were then discussed to make follow-up research directions and recommendations.

## Results

### Quality assessment and risk of bias

In this study, methodological quality was assessed using the Quality Assessment Tool of Systematic Reviews and Meta-Analyses (National Heart Lung Blood Institute, 2019). As shown in Table 1, authors 1 (Li) and 3 (Pan) were satisfied with the methodological assessment of the Chinese literature. Author 2 (Hu) suggested that subsequent studies could include other Chinese databases besides CNKI. In general, the three authors agreed on the assessment of methodological quality.

**Table 1:** Summary of quality assessment and risk of bias.

**Category**	**Author**	**Criteria**								**Quality rating**
		**1**	**2**	**3**	**4**	**5**	**6**	**7**	**8**	
Chinese core journals	Author 1 (Li)	Yes	Yes	Yes	Yes	Yes	Yes	Yes	NA	Good
	Author 2 (Hu)	Yes	Yes	Yes	Yes	Yes	Yes	Yes	NA	Fair
	Author 3 (Pan)	Yes	Yes	Yes	Yes	Yes	Yes	Yes	NA	Good
English core journals	Author 1 (Li)	Yes	Yes	Yes	Yes	Yes	Yes	Yes	NA	Good
	Author 2 (Hu)	Yes	Yes	Yes	Yes	Yes	Yes	Yes	NA	Good
	Author 3 (Pan)	Yes	Yes	Yes	Yes	Yes	Yes	Yes	NA	Good

NA, not applicable; Criteria, 1, Is the review based on a focused question that is adequately formulated and described? 2, Were eligibility criteria for included and excluded studies predefined and specified? 3, Did the literature search strategy use a comprehensive, systematic approach? 4, Were titles, abstracts, and full-text articles dually and independently reviewed for inclusion and exclusion to minimize bias? 5, Was the quality of each included study rated independently by two or more reviewers using a standard method to appraise its internal validity? 6, Were the included studies listed along with important characteristics and results of each study? 7, Was publication bias assessed? 8, Was heterogeneity assessed? (This question applies only to meta-analyses.); You can see the criteria at the following link https://www.nhlbi.nih.gov~health-topics/study-quality-assessment-tools.

The definitive collection of 7 Chinese core-journal articles and 29 English core-journal articles were assessed for content quality through the Quality Assessment Tool for Observational Cohort and Cross-Sectional Studies (National Heart Lung Blood Institute, 2019). As shown in Tables 3, 4, the three authors rated four (57.14%) Chinese core-journal articles as Good and three (42.86%) as Fair, with consistent evaluation results. For English core-journal articles, the three authors agreed that 24 (82.76%) were Good and 5 (17.24%) were Fair.

### Search results

This study adopted the above rigorous and systematic search and screening to initially identify 1,035 Chinese documents and 549 English documents. Through the unified screening criteria, 175 Chinese core-journal articles and 441 English core journal articles were retrieved. Twenty-one Chinese and 42 English core-journal articles focused on school support for the student population were retained after reviewing the titles and abstracts. Through the full-text review, seven Chinese core-journal articles and 29 English core-journal articles that significantly contributed to the school-supported research were finally extracted (Figure 1; Tables 3, 4).

### Study status

#### Research trends

**Figure 2:**
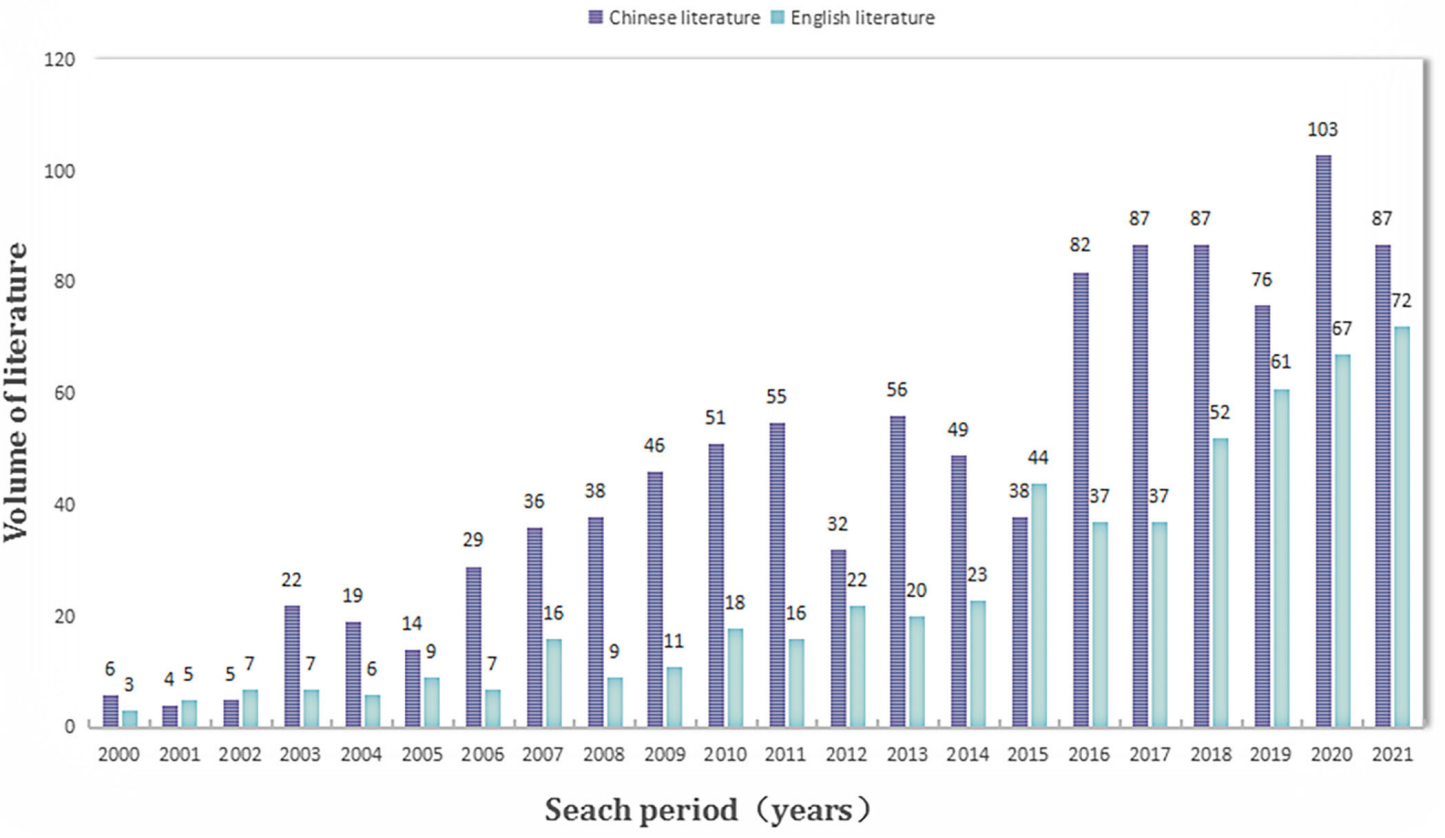
Research trend (*n* = 1,035 for Chinese literature and *n* = 549 for English literature).

To visualize the research trends on school support, 1,035 Chinese literature and 549 English literature were initially identified for statistical analysis. As shown in Figure 2, the number of publications on school support in both Chinese and English showed an overall growth trend from 2000 to 2021, which indicated that both Chinese and Western researchers were paying more attention to school support.

#### Research discipline areas

As shown in Table 2, this study compared the five most common research disciplines in the 1,035 Chinese and 549 English documents initially identified, which identified that research on school support was mainly concentrated in the two disciplines of education and psychology.

**Table 2:** Research discipline areas (top 5).

**Classify**	**CNKI** ***n*** = **1,035**		**WOS** ***n*** = **549**	
	**Discipline areas**	**Volume of documents (Percentage)**	**Discipline areas**	**Volume of documents (Percentage)**
1	Educational theory and management	376 (36.33%)	Educational research	210 (38.25%)
2	Higher education	158 (15.27%)	Psychology	139 (25.32%)
3	Secondary education	127 (12.27%)	Public environmental occupational health	63 (11.48%)
4	Adult and education for special population	121 (11.69%)	Pediatrics	36 (6.56%)
5	Psychology	76 (7.34%)	Psychiatry	24 (4.37%)

The data in the table were collated from the relevant data of CNKI and Web of Science.

#### Study characteristics

Tables 3, 4 present the details of the specific characteristics of seven Chinese and 29 English core journal articles, including basic information, name of the lead author, year of publication, study design and objectives, participants, sample size, research methods, findings, and article quality.

**Table 3:** Summary of included Chinese core-journal articles (*n* = 7).

**References**	**Research design**	**Theoretical bases**	**Research objectives**	**Participants (age range)**	**Sample size**	**Research methodology**	**Measurement tools**	**Findings**	**Article quality**
Luo and Xiang (2011)	Cross-sectional study	None	Exploring school adjustment and school support systems related to students with cerebral palsy	Elementary school students with cerebral palsy (the average age of 10.03 years)	113	Quantitative	Developing the school support system questionnaire for students with cerebral palsy	1. School support consisted of five dimensions, which were environmental support, teacher support, peer support, examination and assessment support, and classroom teaching support. 2. School support had a significant positive predictive effect on the school adjustment for students with cerebral palsy.	Good
Chen (2014)	Case study	None	Exploring the comprehensive influence of school, family and peers on the psychological crisis faced by Chinese college students	College students	15	Qualitative	–	School support, family support and peer support complemented and cooperated with each other to form synergy and produce positive effects on the resolution of the psychological crisis of college students.	Fair
Yang and Li (2016)	Policies study	Positive behavioral support	Exploring school-level positive behavior support in the United States and its implications for China	–	–	Qualitative	–	Positive behavioral support at the school level enabled all students to achieve academic and social development.	Fair
Hu and Liu (2019)	Experimental study	Organizational support theory	1. Designing a mental health intervention study for college students with depression 2. Exploring the differential impact of different types of school support	First-year depressed college students	50	Mixed	–	School support could effectively reduce the negative depression of college students who were prone to depression, and significantly improved the mental health of depressed college students. Compared with peer support, teacher support and friend support had a greater positive impact on depressed students.	Good
Ke et al. (2019)	Cross-sectional study	None	Exploring the status on school resource support from the perspective of students' individualized growth	Elementary school students in grade 5	2,161	Quantitative	Developing a scale of school support with two dimensions of course study resources and venue activity resources	Course study resources and venue activity resources had a high degree of support for students' individualized growth.	Fair
Zhao and Zhou (2019)	Cross-sectional study	Ecological system theory	Exploring the influencing factors of college students' school identity	University students aged 21–23	5,855 (From 35 colleges)	Quantitative	National Survey of Student Engagement in Learning (2014 Edition), USA.	1. School support for college students included 6 kinds of support resources, which were academic support, social interaction support, economic life support, social practice support, health care support, and physical and artistic activity support. 2. School support could significantly enhance school identity.	Good
Zhang et al. (2020)	Longitudinal study	Creativity component theory	1. Exploring the development of creativity in the upper elementary school students 2. Exploring the gender differences in the role of school support	Elementary school students in grades 4–6 (with the average age of 10.43 years)	203	Quantitative	Perceived school climate scale adapted by Jia et al. (2009)	School support was a significant contributor to the development of creativity in the upper primary grades. There were gender differences in perceived school support.	Good

**Table 4:** Summary of included English core-journal articles (*n* = 29).

**References**	**Research design**	**Theoretical bases**	**Research objectives**	**Participants (age range)**	**Sample size**	**Research methodology**	**Measurement tools**	**Findings**	**Article quality**
1. Torsheim et al. (2000) (Norway)	Reliability and validity test of the scale	Social support theory	Presenting results on the factor structure, test-retest reliability, and external validity of the Teacher and Classmate Support Scale, a brief self-report measure on perceived support from teachers and classmates.	Teenagers aged 13–15	681	Quantitative	Developing a scale for measuring teacher support and classmates' support in this study.	The scale offered a parsimonious self-report measure of classmate and teacher support, but more evidence was needed before the scale could be recommended for broader research purposes.	Good
2. Torsheim and Wold (2001) (Norway)	Cross-sectional study	Cognitive activation theory	Examining the relationship between school-related stress, social support from teachers and classmates, and somatic complaints in the general population of Norwegian adolescents.	Students aged 11–16	4,952	Quantitative	1. Social support from teachers was measured with a three-item questionnaire. Items were rated on a 5-point Likert-type scale from strongly agree to disagree strongly. 2. Social support from classmates was measured with a three-item questionnaire. Items were rated on a 5-point frequency scale from always to never.	1. Low classmate support was associated with higher OR of weekly headache and weekly dizziness but not with higher odds for abdominal pain and backache. 2. Low teacher support was associated with higher OR of weekly abdominal pain and weekly dizziness but not with higher OR for weekly headache and backache. 3. The strongest associations of low social support were shown for coexisting symptoms, with an OR of 1.47 for low classmate support and 1.36 for low teacher support.	Fair
3. Chong et al. (2006) (Singapore)	Cross-sectional study	None	Examining the respective contributions of perceived support from parents, peers, and school and the mediating role dispositional optimism plays in these relationships.	Asian adolescents (with the average age of 13.5years)	519	Quantitative	Three scales of the Personality Self-Report (SRP-A) taken from Behavior Assessment System for Children (BASC) were used to measure the adolescents' perceptions of themselves, support received, and their adjustment to the environment.	Positive supportive relationships with parents, peers, and the school were important contextual factors influencing the psychological wellbeing of these adolescents, and dispositional optimism partially mediated support from each of these three sources and psychological adjustment.	Good
4. Jia et al. (2009) (China; USA)	Cross-sectional study	Ecological theories of development	Exploring students' perceptions of 3 dimensions of school climate (teacher support, student-student support, and opportunities for autonomy in the classroom) and the associations between these dimensions and adolescent psychological and academic adjustment in China and the United States.	Students (with the average age of 12.34 years)	1,412	Quantitative	Perceived school climate. The school climate measure used in this study was a revised 25-item version of two school climate measures (Emmons et al., 2002; Brand et al., 2003).	1.Students in China perceived higher levels of teacher support, student-student support, and opportunities for autonomy in the classroom than students in the United States. 2. Students' perceptions of teacher support and student-student support were positively associated with adolescents' self-esteem and grade point average but negatively associated with depressive symptoms for both Chinese and American adolescents.	Good
5. Stadler et al. (2010) (Germany)	Cross-sectional study	School climate theory	Investigating the frequency and effects of peer victimization on mental health problems among adolescents.	Females (with an average age of 14.68 years) and males (with an average age of 14.69 years).	986	Quantitative	School support comprised the composite of three school support scales: Negative school climate (seven items), perceived teacher support (eight items), and attachment to school (five items).	School support was effective in both male and female adolescents by acting as a buffer against the effect of victimization, and school support gained increasing importance in more senior students.	Good
6. Corprew and Cunningham (2011) (USA)	Cross-sectional study	Phenomenological Variant of the Ecological Systems Theory (PVEST)	Exploring the association between negative youth experiences and bravado attitudes in African American urban males. In addition, positive factors, such as school social support, were examined to understand potential resilient pathways.	African American male students aged 13–18	126	Quantitative	The *School Social Support* scale comprised four questions from a more extensive social support scale (Munsch and Blyth, 1993; Munsch and Wampler, 1993).	The results highlighted the importance of adolescent perceptions of support in the school context and how this perceived support may decrease bravado attitudes.	Good
7. Tang et al. (2013) (Taiwan, China)	Cross-sectional study	None	Constructing a model that assesses the effects of school support and self-care behaviors on life satisfaction in adolescents with type 1 diabetes in Taiwan.	Adolescents with T1DM aged 10–18	139	Quantitative	Developing a scale in this study.	School support and self-care behaviors positively influenced adolescents' life satisfaction with type 1 diabetes.	Fair
8. Asikhia Olubusayo and Mohangi (2015) (South Africa)	Interview study	None	Researching the experiences of 11 orphaned adolescents (5 boys and 6 girls aged between 15 and 18 years) affected by HIV and AIDS in a secondary school (in Atteridgeville, Pretoria, South Africa) and the school support provided by them.	Students aged 15–18	11	Qualitative		Participants had a high prevalence of psychological, behavioral, and emotional problems, and the school support provided to them (teacher support, the general school environment, the degree of discrimination, labeling, and bullying in the school) was not insufficient.	Good
9. Cornell et al. (2015) (China)	Cross-sectional study	School climate theory	Examining how school climate theory provides a framework for conceptualizing 2 key features of school climate disciplinary structure and student support that are associated with 3 measures of peer victimization.	Students in grades 7–8	39,364	Quantitative	An eight-item scale was designed to measure the perceived supportiveness of teacher-student relationships with items such as how much they agree that adults in their school “really care about all students” and whether they would seek help from an adult in their school if “another student was bullying me” (Konold et al., 2014).	Higher student support was associated with a lower prevalence of teasing, bullying and general victimization.	Good
10. Babey et al. (2016) (USA)	Cross-sectional study	Social cognitive theory	Exploring the roles of school support, role models, and social participation on adolescent physical activity in racial and income disparities.	Teenagers aged 12–17	2,799	Quantitative	A modified sub-scale of the Resilience Youth Development Module from the California Healthy Kids Survey (Hanson and Kim, 2007; Furlong et al., 2009).	School support might help promote physical activity among Latino, African American, and low-income youth.	Good
11. Bottiani et al. (2016) (USA)	Cross-sectional study	School climate theory	Examining perceptions of school support and variation in perceived caring, equity, and high expectations by student race, school diversity, and socioeconomic context.	Black and white high school students (with the average age of 15.9 years)	19,726	Quantitative	Twelve survey items using a four-point Likert scale were selected from the California Healthy Kids Survey (Hanson and Kim, 2007) and the School Development School Climate Survey (Haynes et al., 2001) to assess school support.	The findings pointed to the need for intervention to improve perceptions of school support for Black youth and all students in lower-income and more diverse schools.	Good
12. Cupito et al. (2016) (USA)	Cross-sectional study	Ecological theory	Examining the relationship between familism and depressive symptoms across relational contexts in adolescence, and whether maternal warmth and support, as well as school support moderated the relationship between familism and depressive symptoms.	Adolescents (with the average age of 14 years)	180	Quantitative	These 23 items were taken from the Child and Adolescent Social Support Scale (CASSS) Version 2 (designed for children from 6th to 12th grade) to measure adolescents' perceived social support from classmates and teachers (Malecki and Demary, 2002).	School support moderated the relationship between familism and adolescent depressive symptoms.	Good
13. Fang et al. (2016) (Canada; Israel)	Cross-sectional study	General Strain Theory	Guiding by the Deterioration Deterrence Model and General Strain Theory, the present study assessed the mediating role of school support and posttraumatic stress (PTS) on two adolescent risk behaviors (i.e., school violence and drug use) among Arab and Jewish Israeli adolescents.	Students in grades 10–11	4,733	Quantitative	The 8-item scale was a sub-scale of an adapted Hebrew version of the California School Climate Survey developed by Furlong (Rosenblatt and Furlong, 1997).	The findings of this study provide evidence for the theorized mediated pathways between political violence exposure and adolescent risk behaviors by posttraumatic stress (PTS) and school support.	Good
14. Strøm et al. (2016) (Norway)	Longitudinal interview study	School climate	Investigating academic performance, absenteeism, and school support amongst survivors of a terrorist attack in Norway.	Students older than 13	490	Mixed		The findings underscored the importance of keeping trauma-exposed students in school and providing support over time.	Good
15. Arslan (2018) (Turkey)	Cross-sectional study	Social Support Theory	Investigating whether social support mediated and moderated the relation between social exclusion and psychological wellbeing at school.	Teenagers aged 11–18	407	Quantitative	1. Social support was measured using the Social and Emotional Health Survey (SEHS; You et al., 2014), which is a 36-item self-report rating instrument developed to measure youths' social and emotional competencies based on the covitality model (Furlong et al., 2014). 2. The SEHS was comprised of 12 sub-scales (three items for each sub-scale) that refer to four latent traits: belief-in-self (self-awareness, self-efficacy, and persistence), belief-in-others (peer support, school support, and family support).	1. Social support sources from family, peers, and school mediated the relationship between social exclusion and youths' psychological wellbeing. Additionally, regression analyses showed that social support also had a moderator role in this association. 2. The role of these resources (family, school, and peer support) varied concerning gender, and the effect of social support was greater in female students.	Good
16. Bennefield (2018) (USA)	Cross-sectional study	Theories of counseling and psychotherapy	Examining the correlates of one measure of psychological wellbeing, positive affect, in the adolescent population, two dimensions of school support (teacher-student relationship and student engagement) and family support (family communication and family closeness) were examined.	Adolescents	10,148	Quantitative	Four questions were used to assess school attachment. Response choices included “Very”, “Somewhat”, “Not very”, “Not at all”.	1. Among the total sample, all dimensions of school and family support measured were correlates of positive affect. When the total sample was divided by gender and race, there were marked differences in the relationship between school and family support across sub-populations. 2. Males and Whites most closely resembled the total sample, while the relationship between dimensions of school and family support was distinct for females and racial and ethnic minorities.	Fair
17. Carney et al. (2018) (USA)	Cross-sectional study	Social Support Theory	Addressing the complexity of relations among bullying perpetrating, victimization, by standing and students perceived school support, acceptance of diversity at school, and perceived school connectedness.	Students in grades 3–6	973	Quantitative	The original perception of support sub-scale of the CAYCI SES (Anderson-Butcher et al., 2013) contained four items, and three aimed at measure support received at school and one targeted for family support.	Bullying perpetrating had direct and indirect negative effects on perceived school support, acceptance of diversity, and school connectedness.	Good
18. Chen et al. (2019) (China; Norway; Sweden)	Cross-sectional study	Ecological system theory	Examining the relationships between social support and boundaries from family, school, and community and student engagement among Chinese adolescents.	Adolescents (with the average age of 14.56 years)	577	Quantitative	Developing a scale in this study.	Family, school, and community support and boundaries were positively related to two dimensions of student engagement (i.e., behavioral and affective.	Fair
19. Ross-Reed et al. (2019) (USA)	Cross-sectional study	**None**	Determining how family, school, peer, and community support influenced rates of violence victimization and self-harm among Gender minority (GM) and cisgender adolescents.	Middle school students	14,188	Quantitative	The 14 resiliency questions were divided into four domains (family, peer, school, and community).	1. School support was associated with lower odds of dating violence and non-suicidal self-injury. 2. There were significant interactions between gender, violence, and support.	Good
20. McCoy et al. (2020) (USA)	Cross-sectional study	Ecological system theory	Understanding the cumulative impact of household dysfunction adverse childhood experiences (ACEs) on adolescent alcohol and marijuana use and examining how family, school, and community support mitigate these relationships.	Middle school studentsfile://C:/Users \alop\ AppData\Local \youdao\dict \Application \ 9.0.1.1\resultui\html \index.html - /javascript:;	26,476	Quantitative	Adolescents were asked a range of questions related to support in school.	1. Results showed that community support moderated the relationship between adverse childhood experiences (ACEs) and alcohol and marijuana use. 2. School support did not moderate the relationship between adverse childhood experiences (ACEs) and alcohol or marijuana use.	Fair
21. Moreira and Lee (2020) (Portugal;USA)	Longitudinal studies	Self-determination theory	Examining the influences of social support from teachers and peers, as well as autonomy support, on students' trajectories of cognitive engagement.	Students aged 6–18	4,054	Quantitative	Students' perceptions of social support at school were measured from two distinct sources: teachers and peers, by two scales from the Portuguese SEI (Moreira et al., 2009).	Cognitive engagement declined over time. This decline was less pronounced in schools where social support from peers and autonomy support was more prevalent.	Good
22. Smith et al. (2020) (USA)	Longitudinal studies	School climate theory	Exploring the relationship between Black students' perceptions of school support for cultural pluralism and perceptions of school climate.	Black teenagers (with an average age of 13.74 years)	336	Quantitative	Four measures captured perceptions of school climate: 1. Psychological Sense of School Membership (PSSM; Goodenow, 1993) for school belonging. 2. The ICS-S (Brand et al., 2003) for teacher-student interactions. 1. The ICS-S (Brand et al., 2003) for student peer interactions. 2. The Psychosocial Climate Scales of the Effective Schools Battery (Gottfredson and Gottfredson, 1999) to measure fairness.	Black youth who rated their school as being supportive of culturally pluralism had more positive ratings of school climate during the following school year after controlling for the previous year's school climate ratings.	Good
23. Yang et al. (2020) (Hong Kong, China)	Cross-sectional study	Self-determination theory	Testing the relationships between peer support, school support, self-determination, and school engagement in 118 secondary school students with special needs integrated into mainstream schools in Hong Kong.	Secondary school students	118	Quantitative	Five items from the Delaware School Climate Survey (general factor; Bear et al., 2011).	1. School support significantly indirectly affected school engagement *via* self-determination as a mediator. 2. The correlations between school support, self-determination, and school engagement were all positive and significant.	Good
24. Delaruelle et al. (2021) (Belgium)	Cross-sectional study	None	Examining the relationship between adolescents' sleep quality and peer, family, and school factors.	Adolescents aged 11–18	8,153	Quantitative	1. Teacher support was defined as the mean score of the following items: I feel that my teachers accept me as I am; I feel that my teachers care about me as a person; I feel a lot of trust in my teachers. 2. Student support consisted of the average score on 3 items: The students in my class enjoy being together; most of the students in my class are kind and helpful; other students accept me as I am. Response options were similar to those for teacher support. 3. School pressure was measured by a single item.	The individual-level results indicated that adolescents' sleep quality was positively related to family support, teacher support, student support, and perceived family wealth.	Good
25. Despoti et al. (2021) (Greece: Cyprus)	Cross-sectional study	Social support theory	Exploring the potential moderating role of perceived social support (school personnel, friends) and gender in the association between distinct psychopathic traits (callous-unemotional traits.	Students aged 9–12	1,442	Quantitative	Social Support was assessed with the 12-item Multidimensional Scale of Perceived Social Support (MSPSS; Zimet et al., 1988) MSPSS consists of three sub-scales, assessing supportive relationships within 3 contexts: family, friends, and school.	1. School and friend perceived social support acted as protective factors against victimization. 2. School and friends perceived social support moderated the link between narcissism and bullying.	Good
26. Esposito et al. (2021) (Italy)	Cross- sectional study	Social support theory	Testing the unique contribution of homophobic victimization on adolescent non-suicidal self-injury (NSSI) and analyzing the buffering role of teachers and classmates' support.	Students aged 13–19	770	Quantitative	This study used the Classroom Life Scale to measure students' perceptions of teachers' and classmates' support (Johnson et al., 1985).	High classmates' support was negatively associated with adolescents' engagement in non-suicidal self-injury (NSSI). Furthermore, higher levels of classmates' support were associated with a lower NSSI frequency only for youth who reported low levels of homophobic victimization.	Good
27. Fredrick et al. (2021) (USA)	Cross-sectional study	School climate theory	Testing whether peer difficulties (specifically social competence and peer victimization) interacted with school support (a component of school climate) in relation to adolescents' sluggish cognitive tempo (SCT) symptoms.	Teenagers aged 13–15	288	Quantitative	The ASCS (Cornell et al., 2015) is a self-report measure of the quality and experience of an authoritative school climate.	1. Adolescent and parent ratings of lower social competence were both associated with higher adolescent-reported sluggish cognitive tempo (SCT) symptoms in the context of low, but not high, school support. 2. Relational and nonphysical victimization was associated with higher self-reported sluggish cognitive tempo (SCT) symptoms in the context of low school support.	Good
28. Parker et al. (2021) (USA)	Interview study	None	Investigating Black adolescents' perceptions of their experiences with COVID-19, including the challenges they encountered, their coping strategies, and their use of religious/spiritual and school-based support.	Black or African American aged 12–17	12	Qualitative		Findings from this research supported calls for mental health providers to employ culturally affirming mental health services and engage in interagency collaboration to support Black youth.	Good
29. Standley and Foster-Fishman (2021) (USA)	Cross-sectional study	Social support theory	Examining the relationship between social support and suicidality among youth from a public health perspective by using (1) a socioecological framework and (2) an intersectional approach to social identity.	Students aged 13–18	5,058	Quantitative	Social support items were derived from the Communities that Care Youth Survey portion of the MIPHY (CTCYS; Arthur et al., 2002). Nine items measured school-level support (e.g., opportunities to engage in activities, provide input at school, and rewards for achievement).	Social support at the family, school, and community levels was significantly associated with lower suicidality scores, and the combination of family and school support was associated with the lowest suicidality scores.	Good

### Theoretical bases and conceptual definition of school support

**Figure 3:**
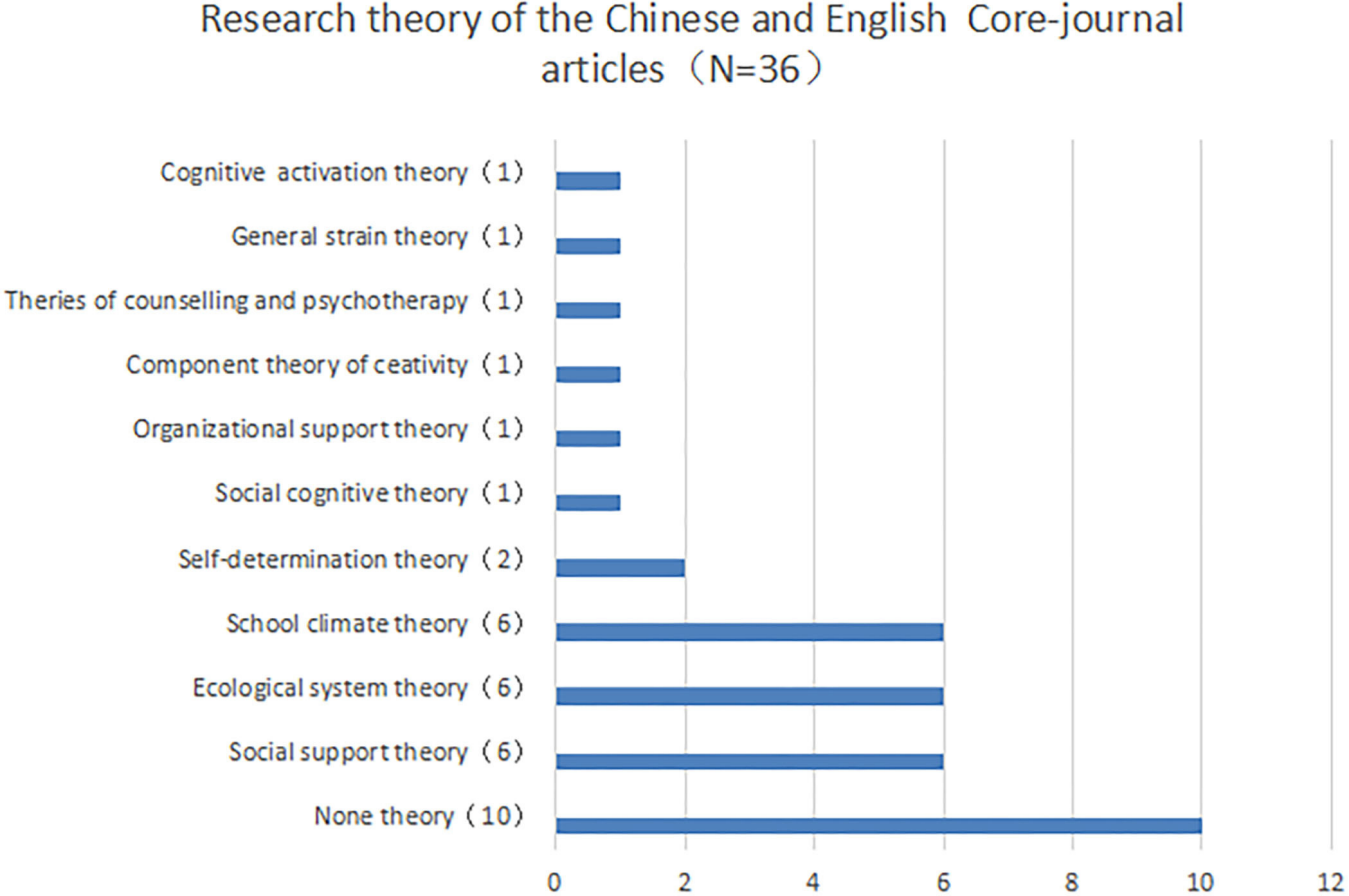
Research theory of the Chinese and English core-journal articles (note: *N* = 36, it is the sum of seven Chinese core-journal articles and 29 English core-journal articles).

Figure 3 presents the research theories of the Chinese and English core-journals articles (listed in Tables 3, 4). The theoretical basis of school support mainly includes social support theory, ecological system theory, and school climate theory.

First, social support theory explains that supportive behaviors that individuals receive or perceive from social relationships have universal meaning and benefits and contribute to an individual's psychological health and positive development (Berkman and Syme, 1979). This theory is widely used, which underlies much of the current research on school support in Chinese and British academic circles. Most studies that draw on social support theory consider school support as a subsystem of the social support system. Teachers and peers at school are the primary sources of social support that influence individual growth. Teacher behavior and support influence the formation of individual values and students' adaptation to the school environment (Chen et al., 2020; Moreira and Lee, 2020). Peer or classmate support is also the main interpersonal support in the social support system, where positive peer relationships can be effective in relieving academic stress and dysphoria (Torsheim and Wold, 2001; Moreira et al., 2018). Therefore, adequate support from teachers and peers can prevent psychological crises (Torsheim et al., 2000; Sun et al., 2021). Research based on social support theory defines school support as the sense of support, security, and recognition that individuals develop in school through interaction with teachers and peers and participation in school activities (Corprew and Cunningham, 2011; Berkowitz and Benbenishty, 2012; Cao, 2016; Moreira and Lee, 2020; Zhang et al., 2020).

Second, ecological system theory emphasizes that individual development is nested within a series of environmental systems that interact with individuals and influence their development, among which microsystems such as family, school, and community are the closest factors that affect individual development (Bronfenbrenner, 1977; Chen et al., 2019; Zhao and Zhou, 2019). Research that draws on ecosystem theory focuses on the environment that supports individual development and emphasizes the interaction of home and school support. Moreover, these studies also demonstrated that school support can mutually complement the role of family support in protecting adolescents' value formation (Cupito et al., 2016). In addition, environmental support provided by family, school, and community can shape an individual's learning cognition, emotion, and behavior (Chen et al., 2019, 2020). Since most studies of school support based on ecosystem theory treat schools as a systematic environmental factor, the conceptual definition of school support is not clearly constructed.

Third, school climate theory holds that school support is a comprehensive reflection of the internal values, school climate, and interpersonal relationships that reflect the quality of school life. Therefore, school climate concept and scale were usually employed to measure school support (Fang et al., 2016; Yang et al., 2020; Fredrick et al., 2021).

### Research methods and participants

**Figure 4:**
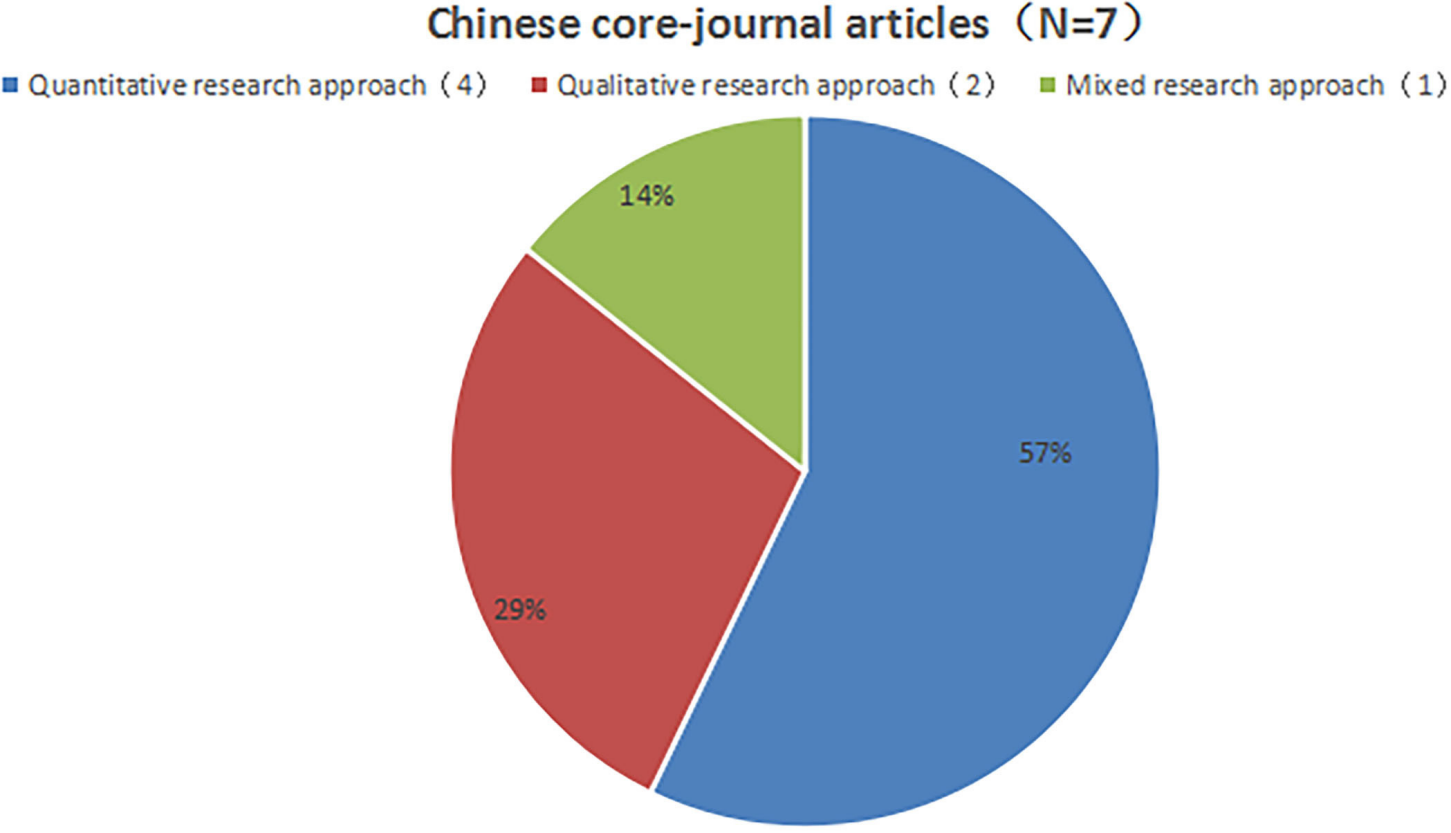
Proportion of research methods in Chinese core-journal articles.

**Figure 5:**
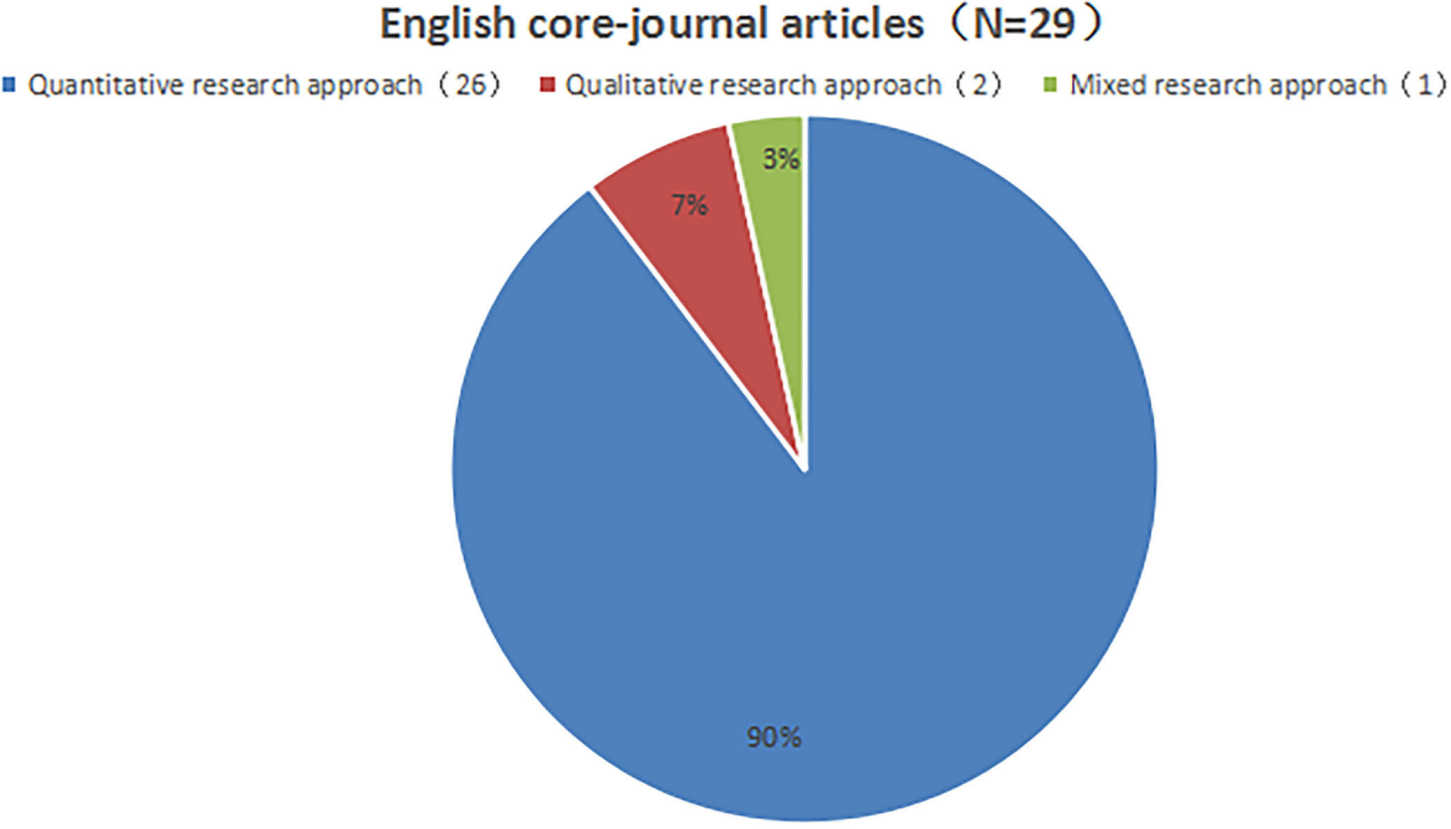
Proportion of research methods in English core-journal articles.

Among the seven Chinese core-journal articles, four were quantitative, two were qualitative, and one was a mixed study (Figure 4). Among the 29 English core-journal articles, 26 were quantitative, two were qualitative, and one was a mixed study (Figure 5). Overall, research on school support mostly employed the quantitative research method.

**Figure 6:**
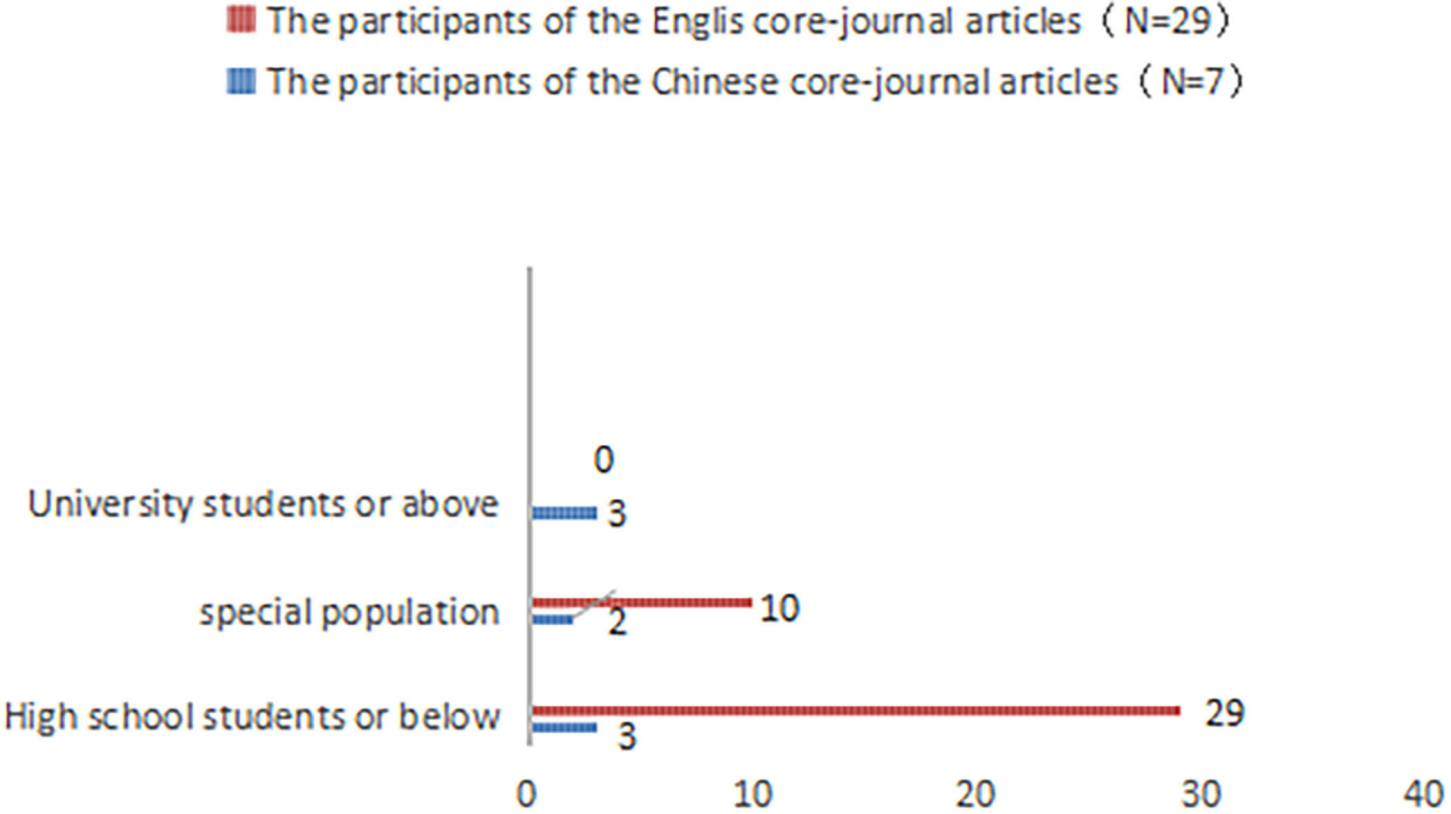
The participants of the Chinese and English core-journals articles (note: some study participants involved both groups, for example, both high school students or below and special groups, so the total number in the figure may be greater than *N*).

As shown in Figure 6, the participants of the seven Chinese core-journal articles (listed in Table 3) were mainly college and primary school students. Luo and Xiang (2011) focused on the effect of school support on the school adaptation of elementary-school students with cerebral palsy. Based on 15 research cases, Chen (2014) explored the comprehensive influence of school, family, and peer groups on Chinese college students facing a psychological crisis. Hu and Liu (2019) conducted an experimental study on school support for college students with depression. Zhao and Zhou (2019) conducted a questionnaire survey on 5,855 college students from 35 domestic colleges and universities and concluded that school support could significantly improve college students' school identities. Ke et al. (2019) developed a scale for school support (including two dimensions of course study resources and venue activity resources) to explore the impact of school support on the personalized growth of 5th-grade students in primary school. Zhang et al. (2020) studied the effects of school support on students in the senior grades of elementary school.

As shown in Figure 6, the participants of the 29 English core-journal articles (listed in Table 4), who mainly were special student groups or groups of students below high school. Torsheim and Wold (2001) conducted a questionnaire on school stress and school support with a sample of 4,952 Norwegian students aged 11–15. In a study of 139 adolescents with diabetes, Tang et al. (2013) found that school support had a significant positive effect on life satisfaction. Asikhia Olubusayo and Mohangi (2015) explored the impact of school support on the psychology and behavior of HIV-infected orphans aged 15–18 through a case study. Through a longitudinal study of survivors who experienced a gun shooting incidence, Strøm et al. (2016) explored the impact of school support on academic performance and absenteeism. Bottiani et al. (2016) focused on the impact of school support on racially diverse high school students. By conducting a study involving 4,733 Israeli high school students, Fang et al. (2016) explored the relationship between youth violence and school support. Carney et al. (2018) tested the hypothesis that bullying is related to school support through a study of 973 elementary school students in grades 3–6 in two public school districts in the northeastern United States.

### Measurement tools of school support

The primary measurement instruments used in the Chinese and English core-journal articles are shown in Tables 3, 4. Most empirical studies use subscales or indicators related to the source of support (such as teacher support, classmate support, or peer support) to measure school support. Measurement items assessed students' responses to statements such as “Overall, adults at my school treat students fairly,” “Students here respect what I have to say,” “My teachers are there for me when I need them,” and “Other students at school care about me” (Torsheim et al., 2000; Litwin, 2001; Torsheim and Wold, 2001; Moreira et al., 2009; Moreira and Lee, 2020; Zhang et al., 2020).

Other studies measured school support using the school climate scale (Bear et al., 2011; Fang et al., 2016; Yang et al., 2020; Zhang et al., 2020). Measurement items assessed students' agreement with statements such as “My teachers care about me,” “Students help one another,” “Students are given the chance to help make decisions,” “Students feel safe at school,” and “School rules are fair to each student.” Bottiani et al. (2016) measured school support in the dimensions of caring, high expectations, and equity by using indicators, such as “My teachers respect students,” “My teachers encourage me to work hard in my class,” and “At this school, students of all races (whether boys or girls and whether parents are rich or poor) are treated the same.”

Chinese researchers (Luo and Xiang, 2011) suggested that school support includes five dimensions, namely environmental support, teacher support, peer support, examination and assessment support, and classroom teaching support. By synthesizing interpersonal support theory and social support theory, Cao (2016) proposed that school support includes three dimensions, namely teacher support, peer support, and student associations support. Based on Tinto's (2012) research on the definition of school support in colleges and universities, Ying (2016) concluded that school support comprised majors and curricular support, teacher teaching support, social support, learning facility support, and living facility support.

## Discussion

### Theoretical implications

First, this study shows the research trend of academic support for schools in Chinese and English academia from 2000 to 2021 through statistical analysis. The results show an overall increasing trend of Chinese and English literature on school support, indicating that more and more researchers pay attention to the importance of school support to adolescents' learning and growth and conduct academic explorations. However, the relevant literature declined in some years, possibly due to a lack of solid theoretical frameworks and measurement tools for school support. According to the research trend chart of the initial search (Figure 2), it can be seen that the number of Chinese literature (*n* = 1,035) seems to be more dominant than the number of English literature (*n* = 549), which may be because the English literature on WOS already belongs to the “core literature.” In contrast, Chinese literature on CNKI includes two categories: non-core journal and core journal. Among them, non-core journal articles were included in the initial steps of identification, which required further screening.

Besides, the identified literature (in both Chinese and English) is concentrated on two disciplines of education and psychology. In other words, the knowledge system of school support involves not only pedagogy but also psychology. Therefore, it is necessary for researchers to integrate a wide range of disciplines to construct a reasonable new system of school support.

Second, this study finds that social support theory, ecosystem theory, and school climate theory are the three theoretical foundations of school support. Other theoretical foundations, shown in Table 3, such as organizational support theory and self-determination theory, remain important for further exploration of school support. There are multiple interpretations of the conceptual definition of school support, each of which has its own advantages. In particular, the seven Chinese core-journal articles lack a clear conceptual definition (Luo and Xiang, 2011; Zhang et al., 2020). Conversely, the concept of school support was clearly defined in the 29 English core-journal articles, which varied according to different research needs (Fang et al., 2016; Strøm et al., 2016; Yang et al., 2020). For example, Fang et al. (2016) agreed with the definition of Berkowitz and Benbenishty (2012) that school support was the degree to which students receive teacher support and a sense of security in school. Yang et al. (2020) defined school support as school values, school climate, and interpersonal relationships that comprehensively reflect the quality of school life. Some scholars defined school support under the particular research background of “school shooting” as sustainable efforts by schools to support traumatized youth (Strøm et al., 2016). With the continuous change and development of new technologies and knowledge systems (Nica, 2018), especially since the outbreak of COVID-19, a large number of studies pointed out that school teaching methods need to be continuously integrated with technology (Erfayliana et al., 2022; Khasawneh, 2022; Pallavi et al., 2022; Warden et al., 2022). At the same time, the concept of school support should also be constantly broadened and deepened. For future related research, whether the concept of school support should be limited to the support of teachers or peers is also topics worth exploring.

Third, the findings indicate that studies in both Chinese and English academia are dominated by quantitative research methods, and there is a lack of qualitative or mixed research methods, especially SLR research. Besides, most of the respondents are special student groups or students below high school, and although three of the seven Chinese core-journal articles have focused on college students, such researches are still very limited. According to this study, schools are important places for adolescents' growth and school support is significant for +academic development, as well as the physical and mental health of all students (Eccles and Roeser, 2011; Tang et al., 2013; Strøm et al., 2016). For college students, higher education is of vital importance for their transformation into society (Padgett et al., 2010). Therefore, it is necessary to pay extensive attention to the school support of college students.

Fourth, the findings discover the lack of internationally recognized scales for measuring school support. The Chinese and English core-journals articles employ or integrate different measurement tools, which are not developed explicitly for school support (Torsheim and Wold, 2001; Yang et al., 2020; Zhang et al., 2020). Specifically, English core-journal articles showed high overlap in measuring social support (Torsheim and Wold, 2001; Cupito et al., 2016; Moreira and Lee, 2020) and school climate (Fang et al., 2016; Yang et al., 2020). For example, scales for teacher support and peer support were integrated or used to measure school support (Torsheim and Wold, 2001). In contrast, Chinese core-journal articles attempted to develop scales for school support but lacked global academic recognition. For example, some researchers (Luo and Xiang, 2011) measured school support from five dimensions, which were environmental support, teacher support, peer support, examination and assessment support, and classroom teaching support, and other researchers (Ke et al., 2019) compiled scales with two dimensions of course study resources and venue activity resources, such as libraries, sports venues, and school buildings. Based on the results, most studies agree that teachers and peers are the two most critical components of school support while the measures of some Chinese studies are more comprehensive.

### Practical implications

First, the findings indicate a possible research space for exploring school support for college students. Considering the irreplaceable positive significance of school support for all students (Sugai and Horner, 2009; Yang and Li, 2016), it is suggested that future research on school support should focus more on college students to enhance the generalizability of the findings.

Second, it reveals that most studies on school support are quantitative and cross-sectional, which suggests that further qualitative or mixed research methods could be employed. Furthermore, it is recommended to conduct more comparative studies in future empirical studies. For example, future researchers can explore the impact of school support on urban migrants and urban children, or on students from different countries or cultural backgrounds.

Third, the results show that among different definitions of school support, most studies use the concept of teacher support or peer support as a substitute for school support. In contrast, school support in a broader sense is less considered. Thus, future research should explore broader aspects, such as whether the resource support on hardware (school libraries, sports venues, and canteen construction) is an aspect of school support.

Finally, this study points to the urgent need to develop new theoretical models and develop specific school support measurement tools. Authoritative and internationally applicable measurement tools are keys to the school's support for the long-term development of research. By further constructing a conceptual system supported by schools and developing scales with better reliability and validity, this topic can be further developed, thus providing a practical reference for educational management.

### Research limitations and future directions for SLR studies on school support

First, only two academic databases (CNKI and WOS) were used in the present study. Future directions regarding SLR studies on school support could be expanded to sample searches in different databases, such as PubMed (US National Library of Medicine, Maryland, USA), Scopus, ScienceDirect (Elsevier, Amsterdam, Netherlands), and Cochrane Library (John Wiley & Sons, New Jersey, USA). Second, this study only selected journals published in Chinese and English. Subsequent SLR studies on school support should take into account publications in other languages. Third, although the risk of bias in methodology and article quality was evaluated, there were only three investigators that had unavoidable personal biases. In follow-up studies, other research methods should be comprehensively considered to reduce bias. Finally, most of the articles in this study were conducted in groups below high school and special student groups, which cannot guarantee a broad representation of the results. Is the positive impact of school support on AIDS orphans aged 15–18 years or on children with cerebral palsy widely applicable to other groups of students with chronic conditions? Does the positive impact of school support on elementary and middle school students apply to college students? The above issues deserve to be further explored in subsequent studies.

## Conclusion

Based on the Chinese and English core-journal articles on research about school support from 2000 to 2021 included in the widely acknowledged academic database CNKI and WOS, this study is the first SLR on school support in Chinese and English academia. Through systematic retrieval and unified screening, seven Chinese core-journals articles and 29 English core-journal articles were retrieved for full-text intensive reading and literature analysis. The results of the study are as follows.

(1) There is an overall upward trend in research on school support.
(2) The two main disciplines are education and psychology.
(3) The theoretical basis of the research is social support theory, ecosystem theory, and school climate theory.
(4) Most of the studies adopt a quantitative approach, and the research objects are mainly focused on special student groups or students below high school.
(5) There is a lack of unified concept and measurement tools for school support.

Relevant studies suggest that school support has obvious positive significance, which deserves further exploration. This study provides a reference for the future development of school support.

## Data availability statement

The original contributions presented in the study are included in the article/supplementary material, further inquiries can be directed to the corresponding author/s.

## Author contributions

JL contributed to the conception of the study and drafted the manuscript. ZH assisted in revising the manuscript. JL, ZH, and LP contributed significantly to the data analysis. LP worked as the writer's assistant. All authors contributed to the article and approved the submitted version.

## Conflict of interest

The authors declare that the research was conducted in the absence of any commercial or financial relationships that could be construed as a potential conflict of interest.

## Publisher's note

All claims expressed in this article are solely those of the authors and do not necessarily represent those of their affiliated organizations, or those of the publisher, the editors and the reviewers. Any product that may be evaluated in this article, or claim that may be made by its manufacturer, is not guaranteed or endorsed by the publisher.

## References

[B1] Amit N. Ismail R. Zumrah A. R. Mohd Nizah M. A. Tengku Muda T. E. A. Tat Meng E. C.. (2020). Relationship between debt and depression, anxiety, stress, or suicide ideation in Asia: a systematic review. Front. Psychol. 11, 1336. 10.3389/fpsyg.2020.01336 32765333 PMC7381269

[B2] Anderson-Butcher D. Amorose A. J. Iachini A. Ball A. (2013). Community and Youth Collaborative Institute School Experience Surveys. Columbus, OH: College of Social Work, The Ohio State University.

[B3] Arslan G. (2018). Social exclusion, social support and psychological wellbeing at school: a study of mediation and moderation effect. Child Indicat. Res. 11, 897–918. 10.1007/s12187-017-9451-1

[B4] Arthur M. W. Hawkins J. D. Pollard J. A. Catalano R. F. Baglioni Jr A. J. (2002). Measuring risk and protective factors for use, delinquency, and other adolescent problem behaviors: the communities that care youth survey. Evalut. Rev. 26, 575–601. 10.1177/0193841X0202600601 12465571

[B5] Asikhia Olubusayo A. Mohangi K. (2015). A case study of school support and the psychological, emotional and behavioural consequences of HIV and AIDS on adolescents : original article. SAHARA J. 12, 123–133. 10.1080/17290376.2015.1125305 26771076

[B6] Axlund McBride R. Lott J. L. (2015). The ecology of volunteerism among college women: identifying campus environments that inform volunteering behaviors. J. Women. High. Educ. 8, 47–65. 10.1080/19407882.2014.987085

[B7] Babey S. H. Wolstein J. Diamant A. L. (2016). Adolescent physical activity: role of school support, role models, and social participation in racial and income disparities. Environ. Behav. 48, 172–191. 10.1177/0013916515609086

[B8] Bear G. G. Gaskins C. Blank J. Chen F. F. (2011). Delaware school climate survey—student: its factor structure, concurrent validity, and reliability. J. Sch. Psychol. 49, 157–174. 10.1016/j.jsp.2011.01.001 21530762

[B9] Bennefield Z. (2018). School and family correlates of positive affect in a nationally representative sample of US adolescents. Child Adolesc Soc Work J. 35, 541–548. 10.1007/s10560-018-0539-3

[B10] Berkman L. F. Syme S. L. (1979). Social networks, host resistance, and mortality: a nine-year follow-up study of Alameda County residents. Am. J. Epidemiol. 109, 186–204. 10.1093/oxfordjournals.aje.a112674 425958

[B11] Berkowitz R. Benbenishty R. (2012). Perceptions of teachers' support, safety, and absence from school because of fear among victims, bullies, and bully-victims. Am. J. Orthopsychiatry 82, 67–74. 10.1111/j.1939-0025.2011.01132.x 22239395

[B12] Bottiani J. H. Bradshaw C. P. Mendelson T. (2016). Inequality in black and white high school students' perceptions of school support: an examination of race in context. J. Youth Adolesc. 45, 1176–1191. 10.1007/s10964-015-0411-0 26746243

[B13] Brand S. Felner R. Shim M. Seitsinger A. Dumas T. (2003). Middle school improvement and reform: Development and validation of a school-level assessment of climate, cultural pluralism, and school safety. J. Educ. Psychol. 95, 570. 10.1037/0022-0663.95.3.570

[B14] Brereton P. Kitchenham B. A. Budgen D. Turner M. Khalil M. (2007). Lessons from applying the systematic literature review process within the software engineering domain. J. Syst. Software 80, 571–583. 10.1016/j.jss.2006.07.009

[B15] Bronfenbrenner U. (1977). Toward an experimental ecological framework. Am. Psychol. 32, 513–531. 10.1037/0003-066X.32.7.513

[B16] Bronfenbrenner U. (1992). Ecological Systems Theory. London: Jessica Kingsley Publishers.

[B17] Cao Y. (2016). A Study of School Support to Immigrated Adolescents Mental Health-Social Work Intervention. Chongqing: Chongqing University. Available online at: https://wap.cnki.net/touch/web/Dissertation/Article/10611-1016731291.nh.html

[B18] Carbia C. López Caneda E. Corral M. Cadaveira F. (2018). A systematic review of neuropsychological studies involving young binge drinkers. Neurosci. Biobehav. Rev. 90, 332–349. 10.1016/j.neubiorev.2018.04.013 29678643

[B19] Carney J. V. Liu Y. Hazler R. J. (2018). A path analysis on school bullying and critical school environment variables: a social capital perspective. Child. Youth Serv. Rev. 93, 231–239. 10.1016/j.childyouth.2018.07.029

[B20] Chen B. B. Wiium N. Dimitrova R. Chen N. (2019). The relationships between family, school and community support and boundaries and student engagement among Chinese adolescents. Curr. Psychol. 38, 705–714. 10.1007/s12144-017-9646-0

[B21] Chen S. M. Zhang W. Li Y. (2020). The effect of parental involvement on child school adaptation: a multiple mediating effects of teacher support and child self-efficacy. Spec. Educ. China 12, 76–82. https://wap.cnki.net/touch/web/Journal/Article/ZDTJ202012012.html (accessed June 28, 2022).

[B22] Chen S. Y. (2014). On the mutual composition of social support between schools, families and peer groups in the mental health of college students - the party building and ideological education in schools. Party Build. Ideol. Educ. Sch. 24, 81–82. Available online at: https://wap.cnki.net/touch/web/Journal/Article/XXDJ201424040.html (accessed June 28, 2022).

[B23] Chong W. H. Huan V. S. Yeo L. S. Ang R. P. (2006). Asian adolescents' perceptions of parent, peer, and school support and psychological adjustment: the mediating role of dispositional optimism. Curr. Psychol. 25, 212–228. 10.1007/s12144-006-1004-6

[B24] Cornell D. Shukla K. Konold T. (2015). Peer victimization and authoritative school climate: a multilevel approach. J. Educ. Psychol. 107, 1186. 10.1037/edu0000038

[B25] Corprew C. S. Cunningham M. (2011). Educating tomorrow's men: perceived school support, negative youth experiences, and bravado attitudes in African American adolescent males. Educ. Urban Soc. 44, 571–589. 10.1177/0013124511406534

[B26] Costa Cordella S. Arevalo Romero C. Parada F. J. Rossi A. (2021). Social support and cognition: a systematic review. Front. Psychol. 12, 637060. 10.3389/fpsyg.2021.637060 33708164 PMC7941073

[B27] Cupito A. M. Stein G. L. Gonzalez L. M. Supple A. J. (2016). Familism and Latino adolescent depressive symptoms: the role of maternal warmth and support and school support. Cult. Divers. Ethn. Minor. Psychol. 22, 517. 10.1037/cdp0000097 27077799

[B28] Delaruelle K. Dierckens M. Vandendriessche A. Deforche B. Poppe L. (2021). Adolescents' sleep quality in relation to peer, family and school factors: Findings from the 2017/2018 HBSC study in Flanders. Qual. Life. Res. 30, 55–65. 10.1007/s11136-020-02614-2 32865698

[B29] Deng Q. Zheng B. Chen J. (2020). The relationship between personality traits, resilience, school support, and creative teaching in higher school physical education teachers. Front. Psychol. 11, 568906. 10.3389/fpsyg.2020.568906 33071897 PMC7541699

[B30] Despoti G. Kokkinos C. M. Fanti K. A. (2021). Bullying, victimization, and psychopathy in early adolescents: The moderating role of social support. Euro. J. Develop. Psychol. 18, 747–764. 10.1080/17405629.2020.1858787

[B31] Eccles J. S. Roeser R. W. (2011). Schools as developmental contexts during adolescence. J. Res. Adolesc. 21, 225–241. 10.1111/j.1532-7795.2010.00725.x

[B32] Eisenberger R. Cummings J. Armeli S. Lynch P. (1997). Perceived organizational support, discretionary treatment, and job satisfaction. J. Appl. Psychol. 82, 812–820. 10.1037/0021-9010.82.5.812 9337610

[B33] Emmons C. L. Haynes N. M. Comer J. P. (2002). The School Climate Survey Revised Edition-Elementary and Middle School Version. New Haven, CT: Yale University Child Study Center.

[B34] Erfayliana Y. Demirci N. Demirci P. T. (2022). Developing online modules for educators in fifth grade physical education class. JUMORA 2, 23–37. 10.53863/mor.v2i1.420

[B35] Esposito C. Affuso G. Amodeo A. L. Dragone M. Bacchini D. (2021). Bullying victimization: investigating the unique contribution of homophobic bias on adolescent non-suicidal self-injury and the buffering role of school support. School Ment. Health. 13, 420–435. 10.1007/s12310-021-09434-w

[B36] Fang L. Schiff M. Benbenishty R. (2016). Political violence exposure, adolescent school violence, and drug use: The mediating role of school support and posttraumatic stress. Am. J. Orthopsychiatry 86, 662. 10.1037/ort0000178 27101429

[B37] Fredrick J. W. Becker S. P. Langberg J. M. (2021). Low school support exacerbates the association between peer difficulties and sluggish cognitive tempo in adolescents. J. Clin. Child Adolesc. Psychol. 1–15. 10.1080/15374416.2021.1923021 34081553 PMC8639839

[B38] Furlong M. J. Ritchey K. M. OBrennan L. M. (2009). Developing norms for the california resilience youth development module: Internal assets and school resources subscales. Calif. School Psychol. 14, 35–46. 10.1007/BF03340949

[B39] Furlong M. J. You S. Renshaw T. L. Smith D. C. O'Malley M. D. (2014). Preliminary development and validation of the social and emotional health survey for secondary school students. Soc. Indicat. Res. 117, 1011–1032. 10.1007/s11205-013-0373-0

[B40] Goodenow C. (1993). The psychological sense of school membership among adolescents: Scale development and educational correlates. Psychol. School. 30, 79–90. 10.1520-6807/(199301)30:1<79::AID-PITS2310300113>3.0.CO;2-X

[B41] Gottfredson G. D. Gottfredson D. C. (1999). Development and Applications of Theoretical Measures for Evaluating Drug and Delinquency Prevention Programs: Technical Manual for Research Editions of What About You (WAY). Gottfredson Associates.

[B42] Gregory A. Cornell D. Fan X. Sheras P. Shih T.-H. Huang F. (2010). Authoritative school discipline: high school practices associated with lower bullying and victimization. J. Educ. Psychol. 102, 483–496. 10.1037/a0018562

[B43] Hanson T. L. Kim J. O. (2007). Measuring Resilience and Youth Development: The Psychometric Properties of the Healthy Kids Survey. Regional Educational Laboratory West.

[B44] Haynes N. M. Emmons C. L. Ben-Avie M. Comer J. P. (2001). The School Development Program Student, Staff, and Parent School Climate Surveys. New Haven, CT: Yale Child Study Center.

[B45] Hopewell S. McDonald S. Clarke M. J. Egger M. (2007). Grey literature in meta-analyses of randomized trials of health care interventions. Cochrane Database Syst. Rev. 1–15. 10.1002/14651858.MR000010.pub3 17443631 PMC8973936

[B46] Hu Y. Liu Z. (2019). Intervention research on mental health of depressed college students: differential effects of different types of school support. J. Educ. Sci. Hunan Normal Univ. 18, 120–125. 10.19503/j.cnki.1671-6124.2019.05.018

[B47] Jia Y. Way N. Ling G. Yoshikawa H. Chen X. Hughes D.. (2009). The influence of student perceptions of school climate on socioemotional and academic adjustment: a comparison of Chinese and American adolescents. Child Dev. 80, 1514–1530. 10.1111/j.1467-8624.2009.01348.x 19765015

[B48] Johnson D. W. Johnson R. T. Buckman L. A. Richards P. S. (1985). The effect of prolonged implementation of cooperative learning on social support within the classroom. J. Psychol. 119, 405–411. 10.1080/00223980.1985.10542911

[B49] Ke H. Xu M. J. Wang J. S. Deng M. (2019). Investigation of the current situation of school resource support from students' personalized growth perspective. J. Shanghai Educ. Res. 11–16. 10.16194/j.cnki.31-1059/g4.2019.11.003

[B50] Khasawneh O. Y. (2022). Technophobia: how students' technophobia impacts their technology acceptance in an online class. Int. J. Hum. Comput. Interact. 1–10. 10.1080/10447318.2022.2085398

[B51] Konold T. Cornell D. Huang F. Meyer P. Lacey A. Nekvasil E.. (2014). Multilevel multi-informant structure of the authoritative school climate survey. School. Psychol. Q. 29, 238–255. 10.1037/spq0000062 24884448

[B52] Litwin H. (2001). Social network type and morale in old age. Gerontologist 41, 516–524. 10.1093/geront/41.4.516 11490050

[B53] Luo L. Xiang Y. Y. (2011). On the relationship between the adaptation to school by students with cerebral palsy and the school supporting system. Chinese J. Spec. Educ. 7, 18–22. Available online at: https://wap.cnki.net/touch/web/Journal/Article/ZDTJ201107006.html (accessed June 28, 2022).

[B54] Malecki C. K. Demary M. K. (2002). Measuring perceived social support: development of the child and adolescent social support scale (CASSS). Psychol. School. 39, 1–18. 10.1002/pits.10004

[B55] McCoy K. Tibbs J. J. DeKraai M. Hansen D. J. (2020). Household dysfunction and adolescent substance use: moderating effects of family, community, and school support. J. Child Adolesc Subst Abuse. 29, 68–79. 10.1080/1067828X.2020.183732026101077

[B56] Moher D. Liberati A. Tetzlaff J. Altman D. Group T. (2009). Preferred reporting items for systematic reviews and meta-analyses: the PRISMA statement. PLoS Med. 6, e1000097–e1000096. 10.1371/journal.pmed.1000097 19621072 PMC2707599

[B57] Moreira P. A. S. Dias A. Matias C. Castro J. Gaspar T. Oliveira J. (2018). School effects on students' engagement with school: academic performance moderates the effect of school support for learning on students' engagement. Learn. Individ. Differ. 67, 67–77. 10.1016/j.lindif.2018.07.007

[B58] Moreira P. A. S. Lee V. E. (2020). School social organization influences adolescents' cognitive engagement with school: the role of school support for learning and of autonomy support. Learn. Individ. Differ. 80, 101885. 10.1016/j.lindif.2020.101885

[B59] Moreira P. A. S. Machado Vaz F. Dias P. C. Petracchi P. (2009). Psychometric properties of the portuguese version of the student engagement instrument. Can. J. Sch. Psychol. 24, 303–317. 10.1177/0829573509346680

[B60] Munsch J. Blyth D. A. (1993). An analysis of the functional nature of adolescents' supportive relationships. J. Early Adolesc. 13, 132–153. 10.1177/0272431693013002001

[B61] Munsch J. Wampler R. S. (1993). Ethnic differences in early adolescents' coping with school stress. Am. J. Orthopsych. 63, 633–646. 10.1037/h0079482 8267104

[B62] National Heart Lung Blood Institute (2019). Study Quality Assessment Tools. Available online at: https://www.nhlbi.nih.gov/health-topics/study-quality-assessment-tools (accessed June 28, 2022).

[B63] Nica E. (2018). The social concretisation of educational postmodernism. Educ. Philos. Theory 50, 1659–1660. 10.1080/00131857.2018.1461364

[B64] Padgett R. D. Goodman K. M. Johnson M. P. Saichaie K. Umbach P. D. Pascarella E. T. (2010). The impact of college student socialization, social class, and race on need for cognition. New Dir. Inst. Res. 2010, 99–111. 10.1002/ir.324

[B65] Pallavi D. R. Ramachandran M. Chinnasamy S. (2022). An empirical study on effectiveness of e-learning over conventional class room learning–a case study with respect to online degree programmes in higher education. Recent Trends Manag. Commer. 3, 25–33. 10.46632/rmc/3/1/5

[B66] Parker J. S. Haskins N. Lee A. Hailemeskel R. Adepoju O. A. (2021). Black adolescents' perceptions of COVID-19: challenges, coping, and connection to family, religious, and school support. Sch. Psychol. 36, 303. 10.1037/spq0000462 34591585

[B67] Putra I. G. N. E. Astell Burt T. Cliff D. P. Vella S. A. John E. E. Feng X. (2020). The relationship between green space and prosocial behaviour among children and adolescents: a systematic review. Front. Psychol. 11, 859. 10.3389/fpsyg.2020.00859 32425867 PMC7203527

[B68] Rosenblatt J. A. Furlong M. J. (1997). Assessing the reliability and validity of student self-reports of campus violence. J. Youth Adolesc. 26, 187–202. 10.1023/A:1024552531672

[B69] Ross-Reed D. E. Reno J. Peñaloza L. Green D. FitzGerald C. (2019). Family, school, and peer support are associated with rates of violence victimization and self-harm among gender minority and cisgender youth. J. Adolesc. Health. 65, 776–783. 10.1016/j.jadohealth.2019.07.013 31564618

[B70] Smith L. V. Wang M. T. Hill D. J. (2020). Black Youths' perceptions of school cultural pluralism, school climate and the mediating role of racial identity. J. School. Psychol. 83, 50–65. 10.1016/j.jsp.2020.09.002 33276855

[B71] Stadler C. Feifel J. Rohrmann S. Vermeiren R. Poustka F. (2010). Peer-victimization and mental health problems in adolescents: are parental and school support protective? Child Psychiatry Human Develop. 41, 371–386. 10.1007/s10578-010-0174-5 20221691 PMC2861171

[B72] Standley C. J. Foster-Fishman P. (2021). Intersectionality, social support, and youth suicidality: a socioecological approach to prevention. Suicide Life threat. Behav. 51, 203–211. 10.1111/sltb.12695 33876493

[B73] Strøm I. F. Schultz J. H. Wentzel Larsen T. Dyb G. (2016). School performance after experiencing trauma: a longitudinal study of school functioning in survivors of the Utøya shootings in 2011. Eur. J. Psychotraumatol. 7, 31359. 10.3402/ejpt.v7.31359 27171613 PMC4864847

[B74] Sugai G. Horner R. H. (2009). Responsiveness-to-intervention and school-wide positive behavior supports: integration of multi-tiered system approaches. Exceptionality 17, 223–237. 10.1080/09362830903235375

[B75] Sun F. Li H. H. Bao J. M. Zen Z. Song W. Jiang S. Y. (2021). The effects of teacher support and peer support on psychological crisis in Chinese adolescents: the mediating role of perceived discrimination. Stud. Psychol. Behav. 19, 209–215. Available online at: https://wap.cnki.net/touch/web/Journal/Article/CLXW202102010.html (accessed June 28, 2022).

[B76] Sutherland S. E. (2004). An introduction to systematic reviews. J. Evid. Based Dent. Pract. 1, 47–51. 10.1016/j.jebdp.2004.02.021

[B77] Tang S. M. Chen S. W. Wang R. H. (2013). Establishing a model to assess the effects of school support and self-care behaviors on life satisfaction in adolescents with type 1 diabetes in Taiwan. J. Nurs. Res. 21, 244–251. 10.1097/jnr.0000000000000008 24241273

[B78] Tinto V. (2012). Completing College: Rethinking Institutional Action. Chicago: University of Chicago Press.

[B79] Torsheim T. Wold B. (2001). School-related stress, school support, and somatic complaints: a general population study. J. Adolesc. Res. 16, 293–303. 10.1177/0743558401163003

[B80] Torsheim T. Wold B. Samdal O. (2000). The teacher and classmate support scale: factor structure, test-retest reliability and validity in samples of 13-and 15-year-old adolescents. Sch. Psychol. Int. 21, 195–212. 10.1177/0143034300212006

[B81] Warden C. A. Yi Shun W. Stanworth J. O. Chen J. F. (2022). Millennials' technology readiness and self-efficacy in online classes. Innov. Educ. Teach. Int. 59, 226–236. 10.1080/14703297.2020.1798269

[B82] Warren J. S. Bohanon Edmonson H. M. Turnbull A. P. Sailor W. Wickham D. Griggs P.. (2006). School-wide positive behavior support: addressing behavior problems that impede student learning. Educ. Psychol. Rev. 18, 187–198. 10.1007/s10648-006-9008-1

[B83] Yang F. Y. Li F. L. (2016). U.S. school-wide positive behavior support (SWPBS): introduction, evaluation & enlightenment. Glob. Educ. 45, 77–84. Available online at: https://wap.cnki.net/touch/web/Journal/Article/WGJN201607009.html (accessed June 28, 2022).

[B84] Yang L. Chiu H. M. Sin K. F. Lui M. (2020). The effects of school support on school engagement with self-determination as a mediator in students with special needs. Int. J. Disabil. Dev. Educ. 69, 399–414. 10.1080/1034912X.2020.1719046

[B85] Ying J. Z. (2016). The Impact of School Supports on Undergraduate Students' Sense of Belonging. Hubei: Huazhong University of Science and Technology. Available online at: https://wap.cnki.net/touch/web/Dissertation/Article/10487-1016780964.nh.html

[B86] You S. Furlong M. J. Dowdy E. Renshaw T. L. Smith D. C. O'Malley M. D. (2014). Further validation of the social and emotional health survey for high school students. Appl. Res. Qual. Life. 9, 997–1015. 10.1007/s11482-013-9282-2

[B87] Zhang J. Fu M. Xin Y. Chen P. Sha S. (2020). The development of creativity in senior primary school students: gender differences and the role of school support. Acta Psychol. Sin. 52, 1057–1070. 10.3724/SP.J.1041.2020.01057

[B88] Zhao B. H. Zhou Y. K. (2019). Factors influencing college students' school identity: a hierarchical linear model analysis. Fudan Education Forum 17, 56–63. 10.13397/j.cnki.fef.2019.04.009

[B89] Zimet G. D. Dahlem N. W. Zimet S. G. Farley G. K. (1988). The multidimensional scale of perceived social support. J. Pers. Assess. 52, 30–41. 10.1207/s15327752jpa5201_2 2280326

